# Respiratory Symptoms and Hamsters or Other Pets: A Large-sized Population Survey in Saitama Prefecture

**DOI:** 10.2188/jea.15.9

**Published:** 2005-04-22

**Authors:** Kyoko Suzuki, Kazunori Kayaba, Tomoko Tanuma, Jun Kitazawa, Hiroshi Yanagawa

**Affiliations:** 1 Graduate School of Health Science, Tokyo Medical and Dental University.; 2 Department of Health and Social Services, Saitama Prefectural University.; 3 Health Promotion Division, Department of Health and Human Services, Saitama Prefecture.

**Keywords:** hamster ownership, respiratory symptoms, population-based study

## Abstract

BACKGROUND: Keeping hamsters as pets has been increasing markedly. Clinical reports have suggested that hamster or other pet ownership is associated with respiratory symptoms. However, this association has not been fully investigated by population-based studies in Japan. The aim of the present study was to investigate the relationship between hamster ownership and respiratory symptoms by using a questionnaire.

METHODS: During the period of August 1 to 20, 2002, we conducted a cross-sectional survey in Saitama Prefecture, which has a total population of approximately 7 million. First, we selected, proportionally to the population size, 100 areas from 5 administration districts of Saitama Prefecture. From each area, 30 households were chosen: 15 living in detached houses and 15 living in other types of dwelling, such as apartment houses. In this way, 2 groups based on type of dwelling (detached house versus other types) could be studied. A lay away plan was carried out. For the survey, 2 questionnaires were developed. One was a questionnaire dealing with household conditions, including pet keeping. The other sought details regarding individual health and lifestyle conditions. The questionnaire dealing with respiratory symptoms asked “whether the respondents had experienced respiratory symptoms (wheezing and/or breathlessness and/or bad cough) in the last 12 months.”

RESULTS: The response rate was 78.9%. There were 7,395 respondents in 2,368 households. There was no association between either dog or cat ownership and respiratory symptoms. In contrast, hamsters kept in the home were positively associated with respiratory symptoms. In a multivariate logistic regression analysis, hamster ownership increased the odds ratio for respiratory symptoms (odds ratio: 1.57; 95% confidence interval: 1.18-2.10).

CONCLUSION: This large size population-survey indicated that hamster ownership is associated with respiratory symptoms.

Several studies have shown that certain pets increase the risk for asthma or other allergic diseases.^1^^-^^5^ In particular, furry pets have been associated with rhinitis and wheezing, and these animals can also provoke allergic reactions.^6^ Some surveys have found that wheezy children were more likely to own a furry pet than non-wheezy children.^3^^,^^4^ However, other surveys have failed to show these associations.^3^^,^^6^^,^^7^ Therefore, it remains unclear whether exposure to pets including hamsters is related to allergic symptoms.

Recently, there has been a notably sharp increase in the number of people who keep hamsters. Among people who kept pets in 1997, 20% kept hamsters.^8^ Some clinical reports have suggested that asthma or respiratory symptoms are causally associated with keeping hamsters and other pets.^8^^-^^14^ Respiratory symptoms in asthma patients who had kept hamsters disappeared after the hamsters were removed.^8^^,^^9^ However, few large population-based studies have been conducted that have explored the association between hamsters and respiratory symptoms relating to allergic respiratory disorders.

The aim of the present study was to investigate the association of pets, especially hamsters, with allergy-related respiratory symptoms in a large-size population-based study.

## METHODS

### Study population

We used data from the cross-sectional survey done in Saitama prefecture, which has a total population of 7 million. In the survey, a stratified-cluster proportional sampling technique was adopted. Initially, 100 areas were randomly selected from 5 administration districts of Saitama prefecture in proportion to their population size.

A lay away plan was used to recruit the households. At the community center in each area, trained interviewers started visiting the closest households to deliver the questionnaires, and afterward collected them during the period of August 1 to 20, 2002. If a household declined to participate in this study, the interviewer moved to the neighboring households until 30 households (15 detached houses and 15 other dwelling types, such as apartments) were recruited. Thus the total sample was made up of 3,000 households.

### Questionnaires

Two questionnaires were developed for the survey. One dealt with household conditions and included questions about pets and the residential environment. The other dealt with the health status and lifestyle of the individual residents. If a family member could not answer the questionnaires for any reason, another family member was to be interviewed instead. The questionnaire regarding pets asked, “Do you have pets?” and then, if ‘yes’, the next multiple-choice question was “What kind of pets do you have: dog, cat, cage bird and/or hamster?” The residential environment questionnaire inquired about the indoor and outdoor environments. The indoor environment questions included exposure to environmental tobacco smoke, type of flooring (carpet or other) and the presence of moisture condensation in summer and winter. Outdoor environment questions dealt with the structural type of the house (wooden or concrete, detached or apartment), and proximity to heavy traffic, a shopping district, a residential district, or a farm. These types of exposures were selected based on reports of previous studies that demonstrated an increased risk of respiratory or allergy symptoms in some of these settings.^15^^-^^24^

Individual residents were asked “Whether you had experienced respiratory symptoms (wheezing and/or breathlessness and/or bad cough) in the last 12 months.” In addition, information regarding sex, age, and smoking habits were obtained by the detailed questionnaire.

### Statistical analysis

The Statistical Package for the Social Sciences (SPSS^®^) version 11.0 for Windows (SPSS Inc., Chicago, IL, USA) was used for all analyses. The number of subjects included in the individual analyses varied slightly due to some missing data.

The association between respiratory symptoms and the characteristics were evaluated by the chi-squared test. Statistical significance is reported at three levels: p<0.05, p<0.01 and p<0.001. A logistic regression analysis was performed to compute adjusted odds ratios with 95% confidence intervals. Age, sex, smoking habits and residential environments were used as confounding factors in the model.

## RESULTS

We received responses from 2,368 households containing 7,395 individuals, ranging in age from under 1 to 95 years. The response rate was 78.9%. However, some households refused to participate in the study or were excluded because their members were absent. Thus the rate quoted above does not include the number of those households in the numerator. Table 1 shows the demographic characteristics, smoking status, pet-ownership status, and the prevalence of respiratory symptoms. Of all individuals, 5,059 (68.0%) reported having a pet, and a hamster was kept by 319 individuals (4.3%).

**Table 1.** Characteristics of the study population.

		n	(%)
Total		7395	
Sex			
	Male	3656	(49.4)
	Female	3730	(50.5)
Age (year)			
	<10	1037	(14.0)
	10-19	853	(11.5)
	20-29	856	(11.6)
	30-39	1325	(17.9)
	40-49	997	(13.5)
	50-59	974	(13.2)
	60-69	799	(10.8)
	69+	546	( 7.4)
Smoking status			
	Current smokers	1844	(24.9)
	Ex-smokers	2634	(35.6)
Pets*	No pets	2336	(31.6)
	Dogs	947	(12.8)
	Cats	456	( 6.2)
	Cage birds	232	( 3.1)
	Hamsters	319	( 4.3)
Prevalence of respiratory symptoms		1065	(14.4)

*: A multiple-choice question was used to ask the kind of pet(s).

Respiratory symptoms were reported by 1,065 respondents (14.4%). The relationships between respiratory symptoms and respondentsﾕ home environments and pet ownership are displayed in Table 2. The prevalence rate of respiratory symptoms was higher in males than in females (p<0.01), and was also higher in individuals less than 20 years of age or over 60 years of age (p<0.001). Current smokers were more likely to have respiratory symptoms than non-smokers (p<0.001). Of note, there was no significant association between respiratory symptoms and exposure to environmental tobacco smoke. Individuals living in houses with concrete and other structures (p<0.05), apartments (p<0.01), had moisture condensation in winter (p<0.001) and were close to a shopping district, heavy traffic, or a farm (p<0.001) were more likely to have respiratory symptoms. There was no statistically significant association between the number of years since the house had been built and the prevalence of respiratory symptoms. Therefore, this variable was excluded from the analysis.

**Table 2.** The prevalence of “respiratory symptoms”: relation to home environment exposures and other questionnaire.

Respiratory symptoms		Prevalence %		P value
Sex				
Male		15.7	(572/3635)	<0.01
Female		13.3	(493/3709)	

Age (year)*				
<20		17.9	(337/1881)	<0.001
20-59		12.4	(513/4137)	
60+		16.2	(215/1331)	

Smoking status				
Current smoker				
	Yes	16.3	(299/1831)	<0.01
	No	13.8	(759/5483)	
Environmental exposure to tobacco smoke^†^				
	Yes	13.8	(394/2860)	n.s.
	No	13.9	(364/2621)	

Present house				
Indoor environments				
Carpet	Yes^‡^	14.9	(724/4871)	n.s.
	No	13.7	(341/2481)	
Moisture condensation (in summer)				
	Yes	17.2	( 59/ 343)	n.s.
	No	14.4	(934/6503)	
Moisture condensation (in winter)				
	Yes	15.2	(880/5788)	<0.001
	No	11.4	(147/1295)	
Pets				
	Yes^§^	15.8	(368/2328)	<0.05
	No	13.9	(693/4981)	

Outdoor environments				
Residence Structure				
	Wooden house	13.6	(498/3670)	<0.05
	Concrete and other	15.4	(549/3570)	
Type				
	Detached	13.4	(531/3975)	<0.01
	Apartment	15.8	(534/3377)	
Living environment				
	Residential district	13.4	(723/5381)	<0.001
	Close to something^¶^	17.5	(261/1495)	

* : The age was divided into three intervals (<20 years, 20-59 years, 60+ years).

† : Analysis was performed for data of never smokers.

‡ : Included using carpet only and others.

§ : Dog, cat, caged bird, hamster, others.

¶ : Close to shopping district, heavy traffic, or farm.

Pet owners reported a higher prevalence of respiratory symptoms than those who did not own pets (p<0.05). As shown in Table 3, there was no association between either dog or cat ownership and respiratory symptoms. In addition, these results remained largely unchanged by whether dogs were kept indoors or out. In contrast, hamsters in the home were positively associated with respiratory symptoms (p<0.001).

**Table 3.** The association between pet ownership and the prevalence of respiratory symptoms.

Pets		Respiratory symptoms	Prevalence (%)	P value
		Yes/Total		
Dogs	Yes	25/945	13.2	n.s.
	No	932/6371	14.6	

Cats	Yes	66/454	14.5	n.s.
	No	994/6882	14.4	

Cage birds	Yes	38/231	16.5	n.s.
	No	1021/7097	14.4	

Hamsters	Yes	68/319	21.3	<0.001
	No	989/7003	14.1	

Based on the above results, we categorized the patients into 3 groups based on age: less than 20 years of age; 20 to 59 years of age; and over 60 years of age. We then used age as a dummy variable in the logistic regression analysis shown in Table 4. After adjusting for the controlling variables, hamster ownership significantly increased the odds ratio for respiratory symptoms (odds ratio 1.57; 95% confidence interval, 1.18-2.10).

**Table 4.** Adjusted odds ratios and their 95% confidence intervals for pet exposure and relation to self-reported respiratory symptoms, controlling for age,* sex, smoking habits, and residential environments.

	odds ratio	95% confidence interval
Dogs	0.99	0.79-1.24
Cats	1.07	0.79-1.45
Birds	1.07	0.72-1.58
Hamsters	1.57	1.18-2.10

*: The age was divided into three intervals (<20 years, 20-59 years, and 60+ years).

## DISCUSSION

In this large-size population survey using a stratified-cluster proportional sampling technique, we found that exposure to hamsters was significantly associated with self-reported respiratory symptoms, while exposure to dogs, cats or birds was not associated with respiratory symptoms. This association remained significant after adjusting for a number of potential risk factors, including indoor environment (environmental tobacco smoke, moisture condensation, or carpet use) and outdoor environment (wooden or concrete structure, detached house or apartment, and proximity to heavy traffic, a shopping district, a residential district and/or a farm).

As a note of caution, extending our findings to the relationship between pets and specific allergic disorders, including bronchial asthma, may not be appropriate, as the subjective scale used could have led to misclassification when discriminating symptoms caused by allergic disorders from those caused by non-allergic conditions. However, a previous study found that self-reported respiratory symptoms were well discriminated from those of asthma diagnosed by physicians.^25^ Having said that, the relationship between respiratory symptoms and allergies in adults seemed to be less significant than of that in children, because non-allergic respiratory diseases, such as chronic obstructive pulmonary diseases, are more prevalent in adults.

Some previous studies have reported that respiratory symptoms or allergic sensitization were associated with exposure to hamsters.^8^^-^^14^ However, these studies did not consider other potential confounding factors such as residential environment, and they surveyed only symptomatic outpatients. Since our study took these factors into account, this allowed us to generalize our finding of an association between hamster raising and respiratory symptoms to the general population.

In our study, allergy-related respiratory symptoms were significantly related to the keeping of hamsters, but not to the keeping of dogs or cats. Several explanations may account for this finding. First, the difference in respiratory symptoms between hamsters and other pets could be caused by a selective bias in favor of having pets. Some studies have reported that parents of allergic children tend not to acquire pets.^3^^,^^6^^,^^7^ It is widely believed that having pet dogs or cats can induce allergic symptoms, so people with allergic symptoms are likely to avoid keeping these pets. On the other hand, the hamster is a new, popular pet whose sales have increased in the last 10 years in Japan.^26^ Therefore, hamster owners might not realize that hamsters play an important role in the development of allergic or respiratory symptoms. Thus, they are less likely to give up hamster ownership, even though they have respiratory symptoms.

A second explanation for the difference in respiratory symptoms between hamsters and other pets may relate to differences in exposure to particular animals as pets in early life. Exposure to pet-attributable allergens in early life can inhibit the development of allergic symptoms.^8^^,^^9^^,^^14^ Some studies have indicated that early exposure to dogs and cats has a protective effect against allergic sensitization and asthma.^27^^-^^34^ Because dogs and cats have been popular pets for a very long time, the inhibiting effect against allergic symptom development may be increased. In contrast, hamster ownership only became popular during the 1990s, so there is little chance of early life exposure or long-term exposure to hamsters. Therefore, keeping pet hamsters might cause respiratory symptoms because the inhibiting anti-allergic effect associated with dogs and cats does not occur.

Finally, people without dogs or cats may still be exposed to dog or cat fur.^34^ It has been reported that antibodies for a component of cat fur have been found in the inhabitants of an island without cats.^36^ Because dogs or cats are kept both indoors and outdoors, people without these pets are often exposed to them in public spaces,^36^^-^^38^ and might acquire some protective effects against allergic symptoms caused by fur. In contrast, hamsters are mostly kept indoors. Thus, there is little opportunity to come in contact with hamsters, and to so induce the protective immunological effects.

This study has several limitations. First, the number of households in which member(s) refused to participate or were absent at the first visit was not available. Therefore, the real response rate could be lower than the above reported rate. The proportion of single households in this study (7.1%) is apparently lower than that in Saitama prefecture in Census 2000 (23.1%). Thus, we most likely failed to collect sufficient data from people who live alone. However, respiratory symptoms were not associated with household composition, and therefore the above problem may not have influenced the results. Second, some items of the questionnaire, such as “close to something” and “heavy traffic,” were insufficient to allow conclusions to be drawn regarding quantitative determination of causality. Third, the time and duration of keeping pets was not considered in this analysis. Thus, the possibility exists that some subjects already had respiratory symptoms before acquiring their pets.

Further examination will be required to determine the causal relationship and underlying mechanisms between hamster ownership and allergic symptoms.

In conclusion, after adjusting for some potential environmental risk factors, we found that the keeping of hamsters is associated with an increased risk of respiratory symptoms based on a large-sized population-based survey in Saitama prefecture. Thus, our results imply a positive association between hamster keeping and respiratory symptoms caused by allergic disorders, as has been suggested by observations in clinical settings. Due to the cross-sectional design of this study, further studies are needed to clarify whether the relationship between hamster keeping and respiratory symptoms is causal.
